# The use of botulinum toxin in head and face medicine: An interdisciplinary field

**DOI:** 10.1186/1746-160X-4-5

**Published:** 2008-03-10

**Authors:** Rainer Laskawi

**Affiliations:** 1 Universitäts-HNO-Klinik, Robert-Koch-Str. 40, D-37075 Göttingen, Germany

## Abstract

**Background:**

In this review article different interdisciplinary relevant applications of botulinum toxin type A (BTA) in the head and face region are demonstrated.

Patients with head and face disorders of different etiology often suffer from disorders concerning their musculature (example: synkinesis in mimic muscles) or gland-secretion.

This leads to many problems and reduces their quality of life. The application of BTA can improve movement disorders like blepharospasm, hemifacial spasm, synkinesis following defective healing of the facial nerve, palatal tremor, severe bruxism, oromandibular dystonias hypertrophy of the masseter muscle and disorders of the autonomous nerve system like hypersalivation, hyperlacrimation, pathological sweating and intrinsic rhinitis.

**Conclusion:**

The application of botulinum toxin type A is a helpful and minimally invasive treatment option to improve the quality of life in patients with head and face disorders of different quality and etiology. Side effects are rare.

## Review

### Historical milestones, introduction

Justinus Kerner first described the symptoms of botulism in detail [1]. Pierre van Ermengem isolated the microorganism "bacillus botulinus" [2]. In 1979 A.B. Scott first used botulinum toxin (BTA) therapeutically to correct strabism injecting the toxin into external eye muscles [3].

The clinical use of BTA expanded during the last years (for review see [4]). A lot of movement disorders and disorders of the autonomous nerve system can be treated with this option (see Table 1) and the head and neck region is an interdisciplinary focus in this field. BTA prevents the release of acetylcholine (ACHE) in synapses. ACHE acts as a neurotransmitter for the innervation of muscles and different gland tissues. Blocking the release of ACHE leads to a reduction of pathological movement of muscles and secretion of glands in the head and neck area increasing the quality of life for patients. In this connection the following pathological states of high interdisciplinary relevance are focused in this article:

**Table 1:** Diseases treated with botulinum toxin type A in head and face medicine with high interdisciplinary relevance

**Movement Disorders**	**Disorders of the Autonomous Nerve System**
Facial nerve paralysis	Hypersalivation, Sialorrhea
Hemifacial spasm	Gustatory sweating, Frey's syndrome
Blepharospasm, Meige-Syndrom	Intrinsic rhinitis
Synkinesis following defective healing of the facial nerve	Hyperlacrimation, Tearing
Support in facial wound healing	
Facial pain syndromes	
	
Oromandibular dystonia	
Palatal tremor	
Bruxism	
Hypertrophy of the masseter muscle	

1. movement disorders of the facial nerve (blepharospasm, hemifacial spasm, facial nerve palsy, synkinesis following defective healing of the facial nerve, aesthetic applications, posttraumatic wound healing preventing excessive scaring),

2. hypersalivation of different etiologies,

3. hyperlacrimation,

4. gustatory sweating and

5. intrinsic or allergic rhinitis.

### Movement Disorders

#### Mimic musculature

Facial nerve paralysis, synkinesis following defective healing of the facial nerve, hemifacial spasm, blepharospasm, aesthetic applications, prevention of scar formation

**Figure 1:**
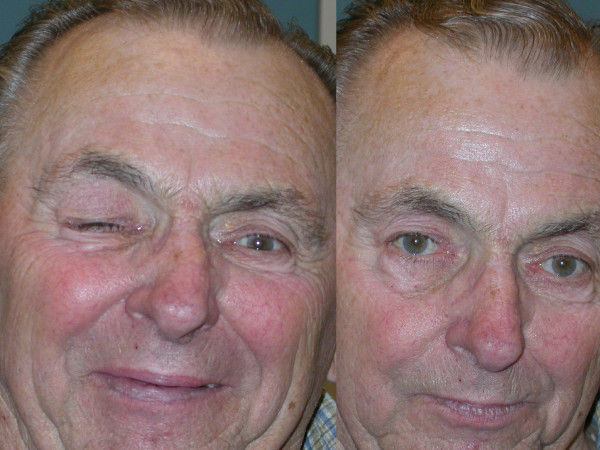
**Left side of the picture: patient with hemifacial spasm on the right side of the face.** He suffers from typical tonic-clonic cramps of the mimic muscles including the frontalis muscle and the platysma. Following BTA injections the face is relaxed and the frequency of tonic-clonic cramps is clearly reduced.

Classical indications to be treated with BTA are the treatment of patients suffering from a *blepharospasm *or a *hemifacial spasm*. Patients with a blepharospasm suffer from repetetive cramps of the orbicularis oculi muscles leading to eye closure. Patients suffering from a hemifacial spasm experience repetetive tonic-clonic cramps of one half of the mimic musculature (example see Fig 1). BTA is suited to treat these diseases by injecting the substance into certain muscle depending on the clinical picture. Doses vary from 1.25 to 5 units Botox^® ^per injection point.

BTA is also helpful in other disorders of the mimic musculature. In some cases a facial nerve paralysis leads to an affection of the cornea with severe problems like a "keratitis e lagophthalmo". In such cases an injection into the levator palpebrae muscle can close the eye for some time to protect the cornea [5]. We use dosages of 5–10 units Botox^®^, the injection is done subcutaneously in the middle of the upper lid. After about 3–4 months the eye "opens" again and that is usually referring to the regeneration time of the paralysis.

In addition the esthetic outcome of a paralysis of the marginal branch of the facial nerve can be improved by injecting 2.5–5 units Botox^® ^into the depressor labii muscle of the normal side [6].

*Synkinesis *are a non-avoidable sequelae following reconstruction of the facial nerve in patients suffering from malignant tumors of the parotid gland. Synkinesis are characterized by synchronous but not intended movements of certain areas of mimic muscles becoming mostly evident during spontaneous movements of the face based on emotional expressions. Mass movements can be reduced using BTA. This options has been described first by our group [7-9].

**Figure 2:**
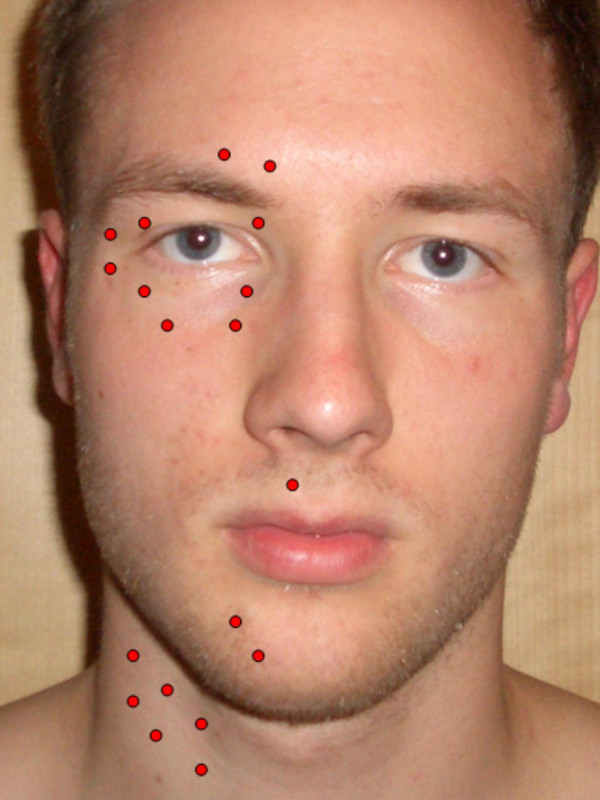
**Typical injection points for pathological movements of mimic muscles.** The dose for each ponit may vary from 1.25 units to 5 units Botox^®^. The number and locations of points depend on the individual character of the disorder in each single patient.

We normally inject BTA using 6 injection-points around the eye to reduce foreign movements of the orbicularis oculi muscle (see Fig 2). The complete injection design and the total dosage depend on the extent of mass movements and may vary from patient to patient. We normally use dosages from 1.25–5 units Botox^® ^on each injection point. Side effects are very rare ; the reason for that could be that lower dosages are necessary to treat synkinesis compared to other facial dyskinesis like hemifacial spasm or blepharospam.

Synkinesis of the *platysma *are of special interest. We focused on this problem and found an acceptable decrease of complaints in treated patients [10,11].

Another interesting indication is the intraoperative application of BTA during the surgical supply of fresh wounds of the face. It has been demonstrated that weakening of face muscles neighbouring facial wounds leads to a better aesthetic outcome. The reason may be that after the immobilization of the treated muscles the borders of fresh wounds better adapt without muscular tension leading to excellent aesthetic results [12].

**Figure 3:**
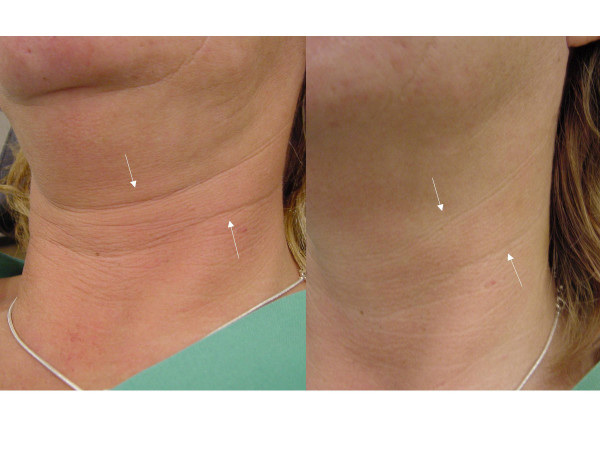
**Effect of BTA on platysma wrinkles: left side: before BTA treatment ; right side: after BTA treatment.** The skin of the neck region is apparently brightened.

The application of BTA to improve the *aesthetic state *of the face is another wide field [13]. Periocular and many other types of wrinkles are in the focus here (example see Fig 3).

**Figure 4:**
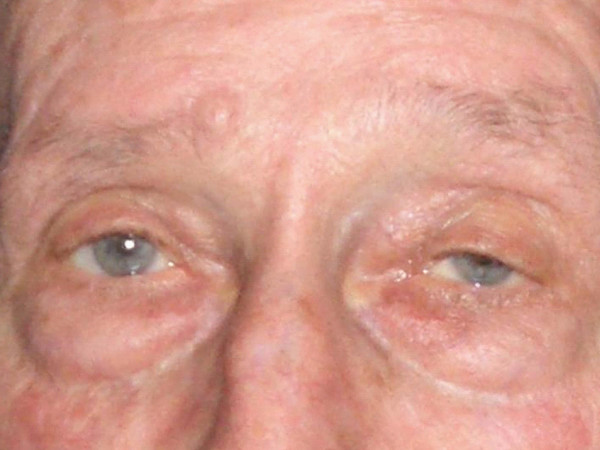
**Patient after BTA treatment of hyperlacrimation on both sides (injection into lacrimal glands).** On the left side (left eye) a mild ***ptosis ***occurred. One can see the difference in the width of the palpebral fissure. The ptosis disappeared within 6 weeks.

For all facial applications the duration of the effect normally begins within 3–5 days and amounts 3–4 months. Then the treatment has to be repeated. Side effects like a ptosis (Fig 4), a "keratitis e lagophthalmo" or tearing are rare.

An important, increasing field of application is the use of BTA in different *pain syndromes*, especially in patients suffering from tension headache, migraine and chronic daily headache (for review see [14,15]).

#### Palatal tremor

Repetitive dystonic contractions of the muscles of the soft palate (*palatoglossus *and *palatopharyngeus *muscles, *salpingopharyngeus*, *tensor *and *levator veli palatini *muscles) lead to a rhythmic elevation of the soft palate [16]. This can cause speech and also swallowing disorders due to a velopharyngeal insufficiency. Most patients suffering from palatal tremor complain of "ear clicking". This rhythmic tinnitus is caused by a repetitive opening and closure of the orifice of the Eustachian tube. A particular sequelae of pathological movements of soft palate muscles is the syndrome of a "patulous Eustachian tube" [17]. These patients suffer from "autophonia" caused by an open Eustachian tube due to the increased muscle tension of the paratubal muscles (*salpingopharyngeus*, *tensor *and *levator veli palatini *muscles).

In a first treatment session, the application of five units of Botox^® ^(uni- or bilaterally) into the soft palate is adequate in most cases. If necessary, this can be increased to two times 15 units of Botox^®^. The application is normally performed transorally (transpalatinal or via postrhinoscopy) under endoscopic control. To optimise the detection of the target muscle, injection under electromyographic control is recommended. To avoid side effects such as iatrogenic velopharyngeal insufficiency the treatment should be started with low doses as described above.

#### Hyperactivity of jaw muscles

##### Oromandibular dystonia (OMD)

**Figure 5:**
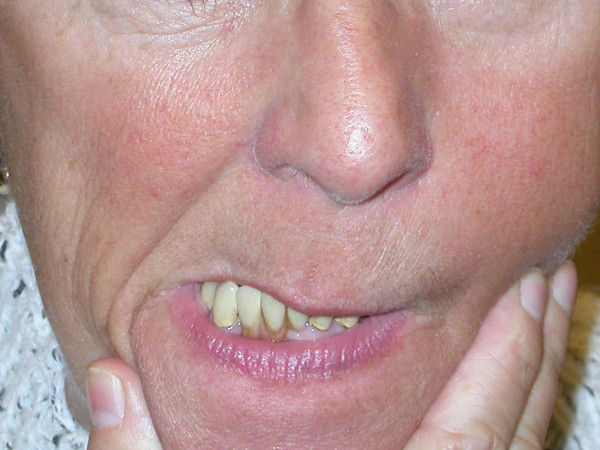
Patient with OMD: Pathologic movements of the mandible are evident, patients use so called "gestes antagonistiques" to break the dystonic activity of the jaw muscles.

In patients with an OMD tongue prostrusions and abnormal movements of the jaw are dominant feature of the clinical picture (Fig 5) [18]. That may result in severe symptoms for the patient like dysarthria and dysphagia. An exact inspection, palpation and electromyographic investigations are suited as diagnostic tools.

Depending of the kind of movement disorder, botulinun toxin injections into the floor of the mouth, the extrinsic tongue muscles and different jaw muscles have to be done to improve the clinical picture. We avoid injections into intinsic tongue muscles because weakening these muscles may result into relevant side effects like swallowing disorders, speech problems and problems of jawing.

Different approaches for injections are described like the external and internal approach of the pterygoideus medialis muscle as an example.

In the treatment of OMD, we use doses up to 50 units Botox^®^.

##### Severe Bruxism [19]

If severe bruxisms does not improve after conventional therapeutic measures, additional injections with botulinum toxin may improve the clinical picture. Injections have to be done into the masseter and temporalis muscles ; doses up to 60 units Botox^® ^per muscle are described. The treatment can be performed using electromyography.

##### Hypertrophy of the masseter muscle

Hypertrophy of the masseter muscle leads to a difference in the symmetry of the face [20].

The injection can be performed transoral or from outside.

In the literature, injections up to 50 units Botox^® ^into each masseter muscle are recommended.

##### Further indications

BTA also is used in patients with a fracture of the jaw for immobilisation of the jaw, in patients with a jaw luxation caused by a hyperactivity of the lateral pterygoid muscle and in patients with a lockjaw.

### Autonomous Nerve System

#### Hypersalivation

**Figure 6:**
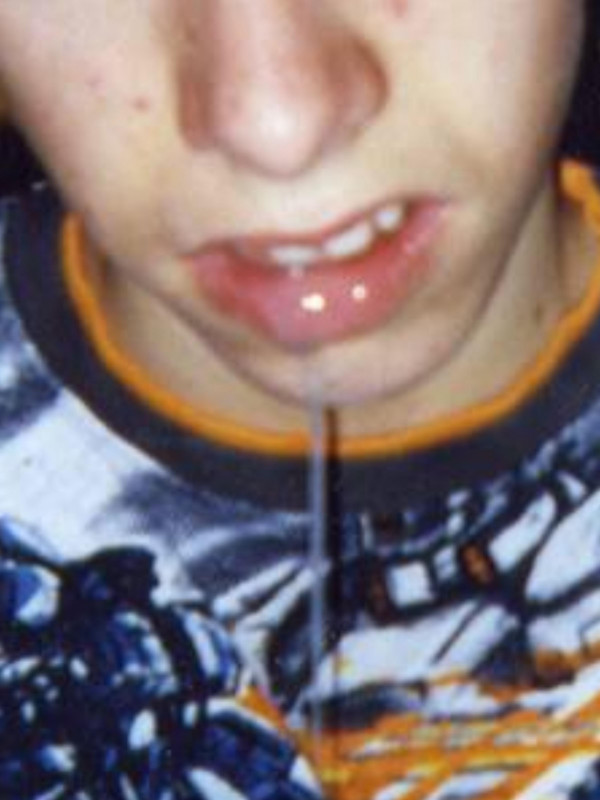
**Patient with extensive hypersalivation (drooling): he is not able to swallow and so looses the saliva out of the mouth.** He suffered from a "herpes-encephalitis" some years ago in his childhood.

Hypersalivation is of high relevance for patients suffering from different diseases (see Fig 6) [21,22]. Some patients of this group are not able to swallow their saliva because of a stenosis of the upper esophagus sphincter region caused by scar formation after a tumor resection. In other patients the sensory control of the entrance of the larynx is reduced and therefore saliva may pass the larynx and reach the trachea and the bronchus. That leads to permanent aspiration and aspiration pneumonia. In a third group of patients problems of the wound healing process after extended surgery exist, like fistulas following laryngectomies. In these cases saliva is a very aggressive agens preventing a normal healing process. In addition, different neurological disorders include hypersalivation as a very serious symptom.

Based on our expanded experiences literature, we prefer in our patients the ultra-sound-guided injection into the parotid and submandibular gland on each side. We inject into the parotid gland 22.5 units Botox^® ^on each side, distributed on 3 points. The submandibular glands are treated by a ultrasound-guided one or 2-point injection of a total of 15 units Botox^® ^per gland. It has been shown by objective datas in a lot of papers that BTA injections are effective in reducing the saliva flow, accompanied by very few side effects.

#### Gustatory sweating, sweating of the face

Gustatory sweating is a common sequelae following parotid gland surgery [23-28]. The treatment of gustatory sweating with BTA has been described first by our group in 1994 (first treated patient December 1993 [23,27]) and became the first line treatment option in these patients.

**Figure 7:**
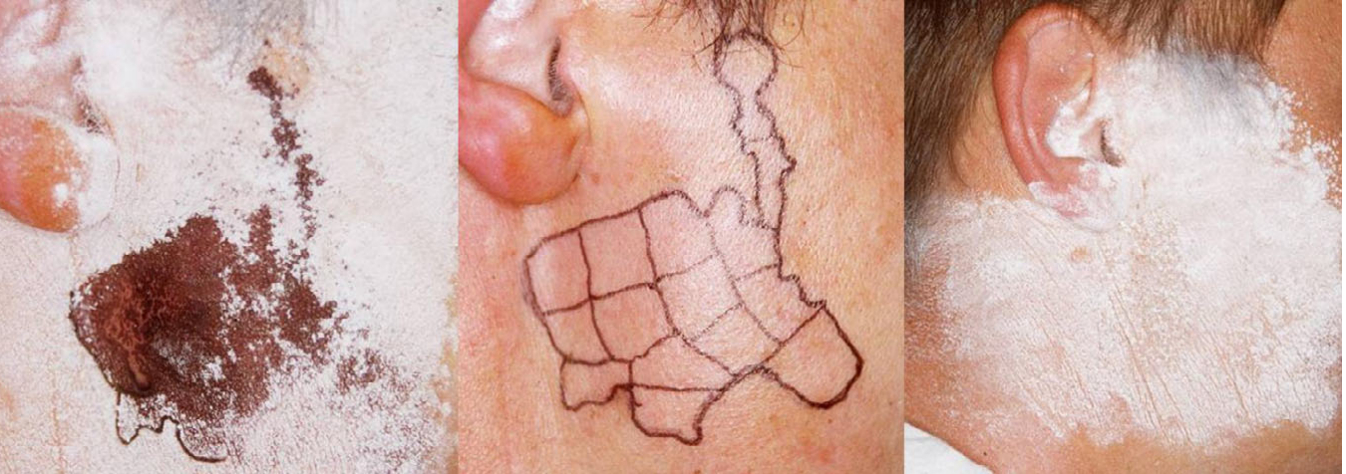
**Patient with gustatory sweating following parotidectomy**. The deep blue color demonstrates the sweating area (left side of picture). The sweating area is marked with a waterproof pen and subdivided in boxes (middle). Following intracutaneous BTA injections, which have to be done intracutaneously, the affected area is completely dry after gustatory stimulation like eating an apple (see right side of picture).

To get an optimal outcome, we recommend marking of the sweating area by Minor's test and then dividing the sweating area in "boxes" using a waterproof pen. The injections are done intracutaneously (see Fig 7).

The effectiveness of BTA treatment in patients with gustatory sweating has been confirmed by a lot of other authors. Some patients report a benefit after BTA-injection already at the same day and interestingly, the positive effect remains much longer than in patients with movement disorders [24]. Some patients reach several years of a symptom free interval.

The treatment of hyperhidrosis of the head and/or the face are based on the same principles as described for patients with gustatory sweating. The doses which are reached for each individual patient depend on the size of the sweating area to be treated.

#### Hyperlacrimation, Tearing

**Figure 8:**
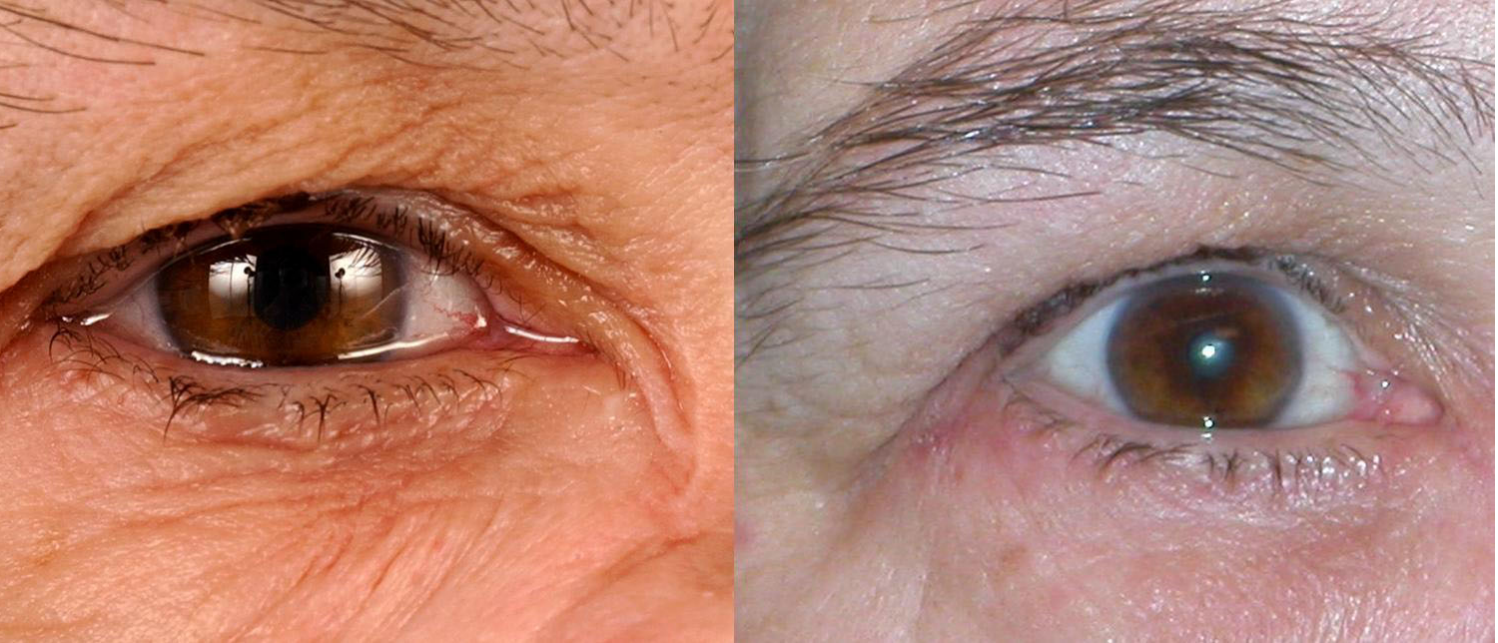
**Patient suffering from a myoepithelial carcinoma of the right maxillary sinus.** After resection of the tumor the transport of tears into the nasal cavity was impossible so that the patient suffered from extensive hyperlacrimation (see left side of picture). Following BTA injection into the pars palpebralis of the right lacrimal gland, hyperlacrimation is reduced but no dry eye occurred (right side). This measure is suited to be done in patients with crocodile tears and any kind of stenosis of the lacrimal duct. It may be a good "interim treatment" before surgery and can be an alternative treatment when patients do not want to undergo surgery.

**Figure 9:**
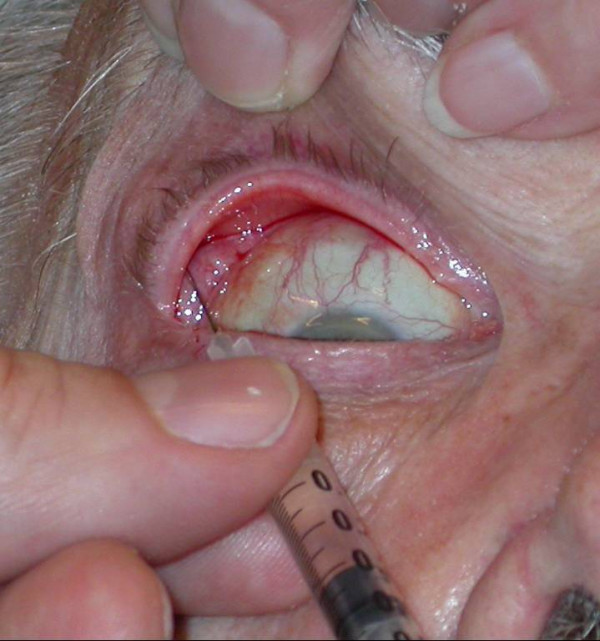
**Application technique of BTA into the right lacrimal gland: A little prominence under the upper lid, which is lifted, up marks the needed direction of the cannula.** Some millimeters after penetrating the tissue and leading the cannula into latero-dorsal direction, the *pars palpebralis *of the lacrimal gland is reached.

Hyperlacrimation (example see Fig 8) can be caused by a stenosis of the lacrimal duct, by misdirected secretory fibers following a degenerative paresis of the facial nerve (crocodile tears) or after a mechanical irritation of the cornea (in patients with a lagophthalmus). The application of BTA is a helpful tool to reduce pathological tearing in these patients in order to reach normal levels of tear liquid production [29-31]. We inject 5–10 units Botox^® ^into the pars palpebralis of the lacrimal gland (technique see Fig 9). None of our patients suffered from a "dry eye" after BTA treatment of the lacrimal gland.

#### Intrinsic Rhinitis

In the last few years, the application of Botulinum toxin type A in patients with intrinsic or allergic rhinitis has been described [32-34]. In experimants the existence of apoptosis of nasal glands has been demonstrated [33]. The main symptom in patients suffering from these diseases is extensive rhinorrhea with secretions dripping from the nose.

**Figure 10:**
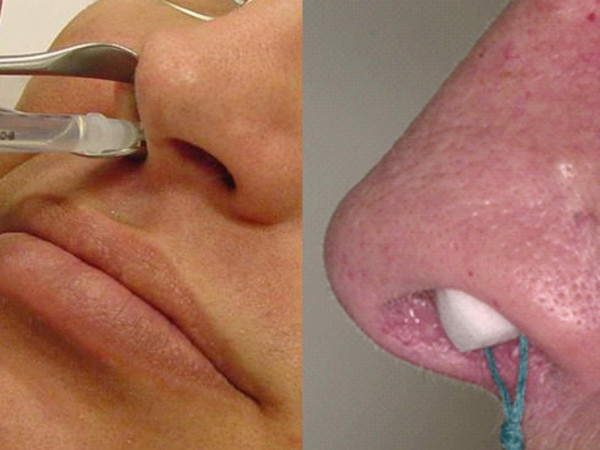
**Technique of BTA application in patients with intrinsic or allergic rhinitis.***Left side of the picture*: injection into the lower or middle turbinate. *Right side of the picture *(sponge technique): A sponge is placed into the nasal cavity and then filled up with BTA solution. After filling, the sponge expands and comes into close contact with the nasal mucosa. The sponge remains located in the nasal cavity for 45 minutes

There are two methods for applying BTA in these patients (Fig 10): it can either be injected into the middle and lower nasal turbinates [32], or it can be applied with a sponge soaked in a solution of BTA (Fig. 10) [34].

For the injection we use 10 units of Botox^® ^for each turbinate (middle and lower nasal turbinates).

With the other technique, the sponges are loaded with a solution containing 40 units of Botox^® ^and one is applied on each side.

**Figure 11:**
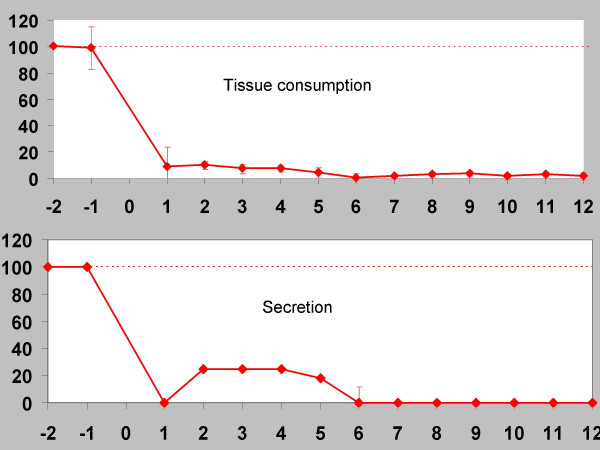
**Patient example, intrinsic rhinitis (*ordinate*: time/weeks ; *abscissa*: per cent (%) of pre-treatment average values (for *tissue consumption*: number of used tissues, for *secretion*: subjective patient's scoring from 1–4) : After BTA treatment (sponge technique) a dramatic improvement of symptoms is to recognize.** Sneezing (A), tissue consumption (B) and nasal secretion (C) are clearly reduced. The documentation of the parameters mentioned begins 2 weeks before BTA treatment in a so called "nose diary".

The positive effect of the injections has been demonstrated in placebo-controlled studies [32]. Nasal secretion is reduced for about 12 weeks (example see Fig 11). Side effects such as epistaxis or nasal crusting were uncommon.

#### New developments and aspects

Some new developments in the use of BTA in head and face medicine are to mention here (see [35]). BTA application in patients suffering from *tinnitus *[36] or *depressions *[37] have been treated with BTA. Further investigations will show whether there is a real hope for clinical use of BTA in these indications.

## Conclusion

The application of botulinum toxin type A is a helpful and minimally invasive treatment option in different functional disorders improving the quality of life in patients with head and face disorders of different etiology. Side effects are rare.

## Abbreviations

BTA: botulinum toxin; ACHE: acetylcholine; OMD: oromandibular dystony.

## Competing interests

The author(s) declare that they have no competing interests.

## Authors' contributions

The author issued the whole manuscript.

## Consent

It is stated that informed written consent was obtained for publication of the patients images.
